# Supplementary material for: Susceptibility of the Different Oxygen-Sensing Probes to Interferences in Respirometric Bacterial Assays with Complex Media
Source: Sensors (Basel). 2024 Jan 2;24(1):267. doi: 10.3390/s24010267 (PMC10781214; doi:10.3390/s24010267)
Supplement: Supplementary file 1 [file sensors-24-00267-s001.zip › sensors-2733214-supplementary.pdf]

Supplementary Information

Susceptibility of the Different Oxygen-Sensing Probes to Interferences in Respirometric Bacterial Assays with Complex Media

Chiara Zanetti [1], Liang Li [1], Rafael Di Lazaro Gaspar [2], Elisa Santovito [3], Sophia Elisseeva [1], Stuart G. Collins [4], Anita R. Maguire [4] and Dmitri B. Papkovsky [1,*]

[1] School of Biochemistry and Cell Biology, University College Cork, Pharmacy Building, College Road, T12 K8AF Cork, Ireland; czanetti@ucc.ie (C.Z.); liang.li@ucc.ie (L.L.)

[2] Cell Analysis Division, Agilent Inc., Euro House, Little Island, T45 WK12 Cork, Ireland; rafael.gaspar@agilent.com

[3] National Research Council of Italy, Institute of Sciences of Food Production, Via Amendola 122/O, 70126 Bari, Italy; elisa.santovito@ispa.cnr.it

[4] School of Chemistry, University College Cork, Pharmacy Building, College Road, T12 YN60 Cork, Ireland; stuart.collins@ucc.ie (S.G.C.); a.maguire@ucc.ie (A.R.M.)

* Correspondence: d.papkovsky@ucc.ie; Tel.: +353-21-490-1698

Table S1. Composition of the different media.

Selective Media	Ingredient	Concentration
Nutrient Broth (NB)	"Lab-lemco" Powder	1.0 g/L
	Peptone	5.0 g/L
	Sodium chloride	5.0 g/L
	yeast extract	2.0 g/L
MacConkey (MC)	Bile salts	5.0 g/L
	neutral red	0.075 g/L
	Peptone	20.0 g/L
	Sodium chloride	5.0 g/L
M-Lauryl Sulfate (MLS)	phenol red	0.2 g/L
	sodium lauryl sulfate	1 g/L
	yeast extract	6 g/L
	lactose	30 g/L
Rapid Coliform ChromoSelect (RCC)	X-gal	0.08 g/L
	MUG (4-Methylumbelliferyl a	0.05 g/L
	IPTG	0.1 g/L
	Peptone	5 g/L
	dipotassium hydrogen phospha	2.7 g/L
	potassium dihydrogen phospha	2 g/L
	sodium lauryl sulfate	0.1 g/L
	sorbitol	1 g/L
	sodium chloride	5 g/L
Minerals Modified Glutamate (MMG	bromocresol purple	0.02 g/L
	ferric ammonium citrate	0.02 g/L
	magnesium sulfate	0.2 g/L
	nicotinic acid	0.002 g/L
	lactose	20 g/L
	pantothenic acid	0.002 g/L
	sodium formate	0.5 g/L
	calcium chloride	0.02 g/L
	L-cystine	0.04 g/L
	dipotassium phosphate	1.8 g/L
	L-arginine	0.04 g/L
	L-aspartic acid	0.048 g/L
	thiamine	0.002 g/L

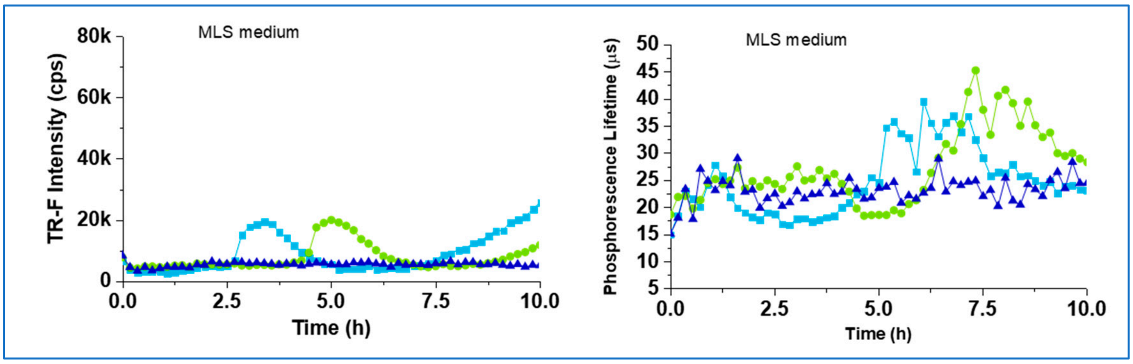

Figure S1. Blank-corrected respiration profiles of the Int and LT signals for E. coli cells in MLS media, produced by PtGlc4 shown in Figure 3E.

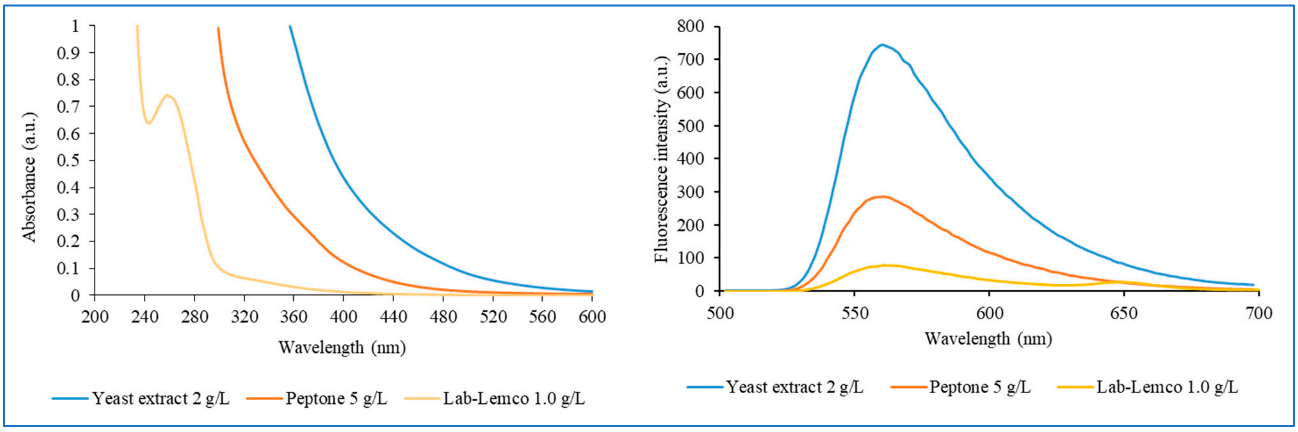

Figure S2. Absorption and fluorescence spectra of yeast extract (2.0 g/L), peptone (5.0 g/L) and Lab-Lemco (1.0 g/L) in PBS buffer.
